# RNA-seq analysis of lignocellulose-related genes in hybrid Eucalyptus with contrasting wood basic density

**DOI:** 10.1186/s12870-018-1371-9

**Published:** 2018-08-06

**Authors:** Katsuhiko Nakahama, Nobuaki Urata, Tomotaka Shinya, Kazunori Hayashi, Kazuya Nanto, Antonio C. Rosa, Akiyoshi Kawaoka

**Affiliations:** 1 Nippon Paper Industries Co., Ltd., Agri-Biotechnology Research Laboratory, 5-21-1 Oji, Kita-ku, Tokyo, 114-0002 Japan; 2 Forest Research Division, Amapá Florestal e Celulose S.A, Rua Claudio Lucio Menteiro, S/N, Santana, Amapa 68925-000 Brazil; 3 Present address: Akita-Jujo Chemicals Co., Ltd., 1-1 Araya-Torikimachi, Akita, 010-1633 Japan

**Keywords:** Hybrid Eucalyptus, Wood basic density, Cellulose, Hemicellulose, Lignin, RNA-Seq

## Abstract

**Background:**

Wood basic density (WBD), the biomass of plant cell walls per unit volume, is an important trait for elite tree selection in kraft pulp production. Here, we investigated the correlation between WBD and wood volumes or wood properties using 98 open-pollinated, 2.4 to 2.8 year-old hybrid Eucalyptus (*Eucalyptus urophylla* x *E. grandis*). Transcript levels of lignocellulose biosynthesis-related genes were studied.

**Results:**

The progeny plants had average WBD of 516 kg/m^3^ with normal distribution and did not show any correlations between WBD and wood volume or components of α-cellulose, hemicellulose and Klason lignin content. Transcriptomic analysis of two groups of five plants each with high (570–609 kg/m^3^) or low (378–409 kg/m^3^) WBD was carried out by RNA-Seq analysis with total RNAs extracted from developing xylem tissues at a breast height. Lignocellulose biosynthesis-related genes, such as cellulose synthase, invertase, cinnamate-4-hydroxylase and cinnamoyl-CoA reductase showed higher transcript levels in the high WBD group. Among plant cell wall modifying genes, increased transcript levels of several expansin and xyloglucan endo-transglycosylase/hydrolase genes were also found in high WBD plants. Interestingly, strong transcript levels of several cytoskeleton genes encoding tubulin, actin and myosin were observed in high WBD plants. Furthermore, we also found elevated transcript levels of genes encoding NAC, MYB, basic helix-loop-helix, homeodomain, WRKY and LIM transcription factors in the high WBD plants. All these results indicate that the high WBD in plants has been associated with the increased transcription of many genes related to lignocellulose formation.

**Conclusions:**

Most lignocellulose biosynthesis related genes exhibited a tendency to transcribe at relatively higher level in high WBD plants. These results suggest that lignocellulose biosynthesis-related genes may be associated with WBD.

**Electronic supplementary material:**

The online version of this article (10.1186/s12870-018-1371-9) contains supplementary material, which is available to authorized users.

## Background

Woody biomass is an important resource because of its sustainability and it is utilized in several ways, such as pulp, paper, civil construction, furniture and energy production. In commercial forest plantations, wood production has increased by silvicultural management and conventional tree breeding programs. However, improving tree growth rates and wood quality still remains a major challenge. Wood quality is determined by the three major components of wood (cellulose, hemicellulose and lignin), physical properties such as microfibril angle, fiber length and wood basic density (WBD). WBD is the biomass of plant cell walls per unit volume and is an important trait for the estimation of biomass and carbon stocks from tree stems [1]. WBD is one of the most important indicator for wood quality as well as for kraft pulp yield for Japanese pulp and paper industries that import wood chips from abroad. Several reports have demonstrated that WBD is under moderate genetic control, suggesting WBD could be improved by selection of traits within the tree breeding program. The growth rates of trees affect WBD and tracheid properties [2, 3]. Thus, silvicultural practices that increase tree growth may cause changes in wood properties as these are related to the relative amounts of different cell types present as well as to their properties [4–6]. For example, in Jack pine and Norway spruce trees grown on mineral soils, increased tree growth via thinning usually decreases WBD [7, 8], a decline that is due to the accelerated production of earlywood relative to latewood. Plant cell walls are mainly composed of cellulose, hemicellulose and lignin, which are found in tracheary elements (tracheids in seedless vascular plants and gymnosperms, and vessels in angiosperms) and fibers in the primary xylem and the secondary xylem (wood) in angiosperms. Their function is to provide mechanical strength to these cell types, which serve as mechanical tissues that facilitate growth of vascular plants to great heights. Lignin deposition in tracheary elements not only reinforces these water conduits to oppose the negative pressure through transpiration, but also renders them hydrophobic for efficient water transport. Cellulose is a fundamental structural unit that has microfibrils cross-linked with hemicellulose to form the framework. The thickness of plant cell walls depends on the harmonization of these three components. Lignin makes the cellulose and hemicellulose networks to provide additional mechanical strength, rigidity and hydrophobicity to the plant cell wall.

**Fig. 1:**
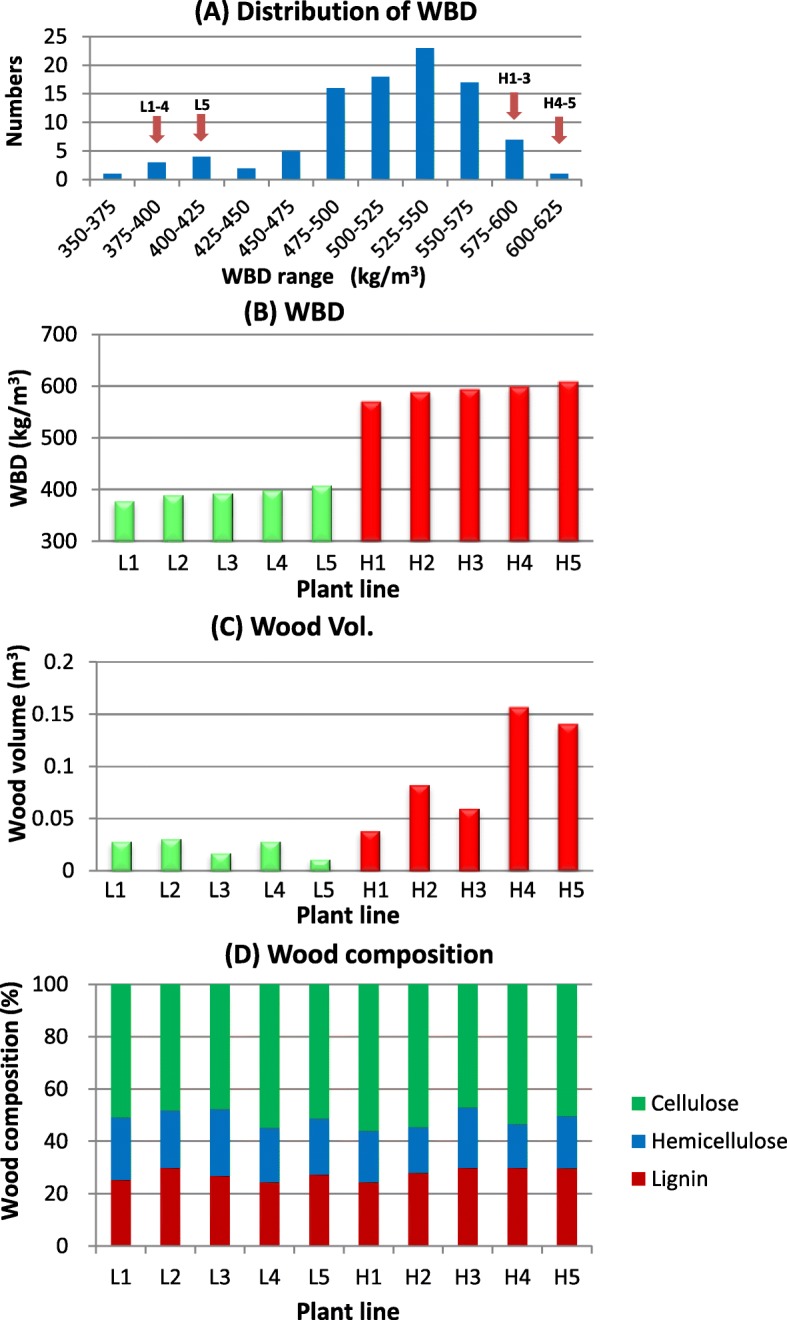
Distribution of WBD in progeny plants (**a**), comparison of WBD (**b**), wood volume (**c**) and wood composition (**d**) between the ten selected genotypes (L1–5 and H1–5)

With regards to the molecular mechanism involved in WBD, several reports have analyzed the transcript levels in *Eucalyptus* species, its hybrid, and in *Pinus radiata* [2, 9, 10]. These reports aimed to identify candidate genes involved in WBD based on Quantitative trait locus (QTL) analysis and they did not provide enough data on gene expression of lignocellulose biosynthesis. In this study, we investigated the WBD of 98 progeny plants of a F1 mother tree that had 587 kg/m^3^ of WBD at 3.7 years of age by open pollination, and selected five plants in each of the two groups- high or low WBD, exhibiting 570–609 kg/m^3^ or 378–409 kg/m^3^ WBD, respectively (Fig. 1). We then compared the transcript profiles of lignocellulose biosynthesis-related genes in developing xylem tissues of these two groups. This experimental design is based on a bulked segregant analysis [11]. The progeny plants were evaluated in seed-grown seedlings generated from parent trees that had suitable traits such as growth rate, WBD or kraft pulp yield. Here, we carried out the correlational analysis between transcript levels of lignocellulose biosynthesis-related genes and WBD in plants with low and high WBD content. Most lignocellulose biosynthesis related genes exhibited a tendency to transcribe at relatively higher level in high WBD plants. We also found that several transcription factors, such as NAM, ATAF, and CUC (NAC), Myeloblastosis (MYB), basic helix-loop-helix (bHLH), homeodomain (HD), WRKY and Lin-11, Isl-1, Mec-3-like (LIM), may act as positive or negative regulators for WBD. The results in this study suggest that lignocellulose biosynthesis-related genes may be associated with WBD.

## Methods

### Plant materials

We used Clone A (6.0 year-old), which showed high WBD (607 kg/m^3^) at the experimental plantation field in Amapá Florestal e Celulose S.A., Amapa state, Brazil (4.97° N, 48.78°W, 40 m elevation) and investigated the correlation between WBD and transcript levels of its open-pollinated progeny. Clone A was *E. urophylla* x *E. grandis* F1 hybrid and was used as the mother tree of these progeny plants, but fathers were not identified. Pollens could have been contributed by the same plant from self-pollination. Ninety-eight 2.4 to 2.8 year-old trees were assessed for Pylodyn penetration (3 penetrations per tree at 1.2 m height) and five *Eucalyptus* progeny genotypes each (*E. grandis* x *E. urophylla*) with contrasting WBD were selected (Fig. 1).

### Simple sequence repeat (SSR) analysis

Genomic DNAs of ten selected progeny plants were extracted from leaf or cambium tissue samples by DNasy Plant mini Kit (QIAGEN, Hilden Germany). In order to identify pollen parents, SSR analysis was carried out using EMBRA primers 15, 38, 78, 95, 117 and 229 [12]. The PCR amplification conditions were: 94 °C for 5 min, followed by 30 cycles of 94 °C for 1 min, the primer specific annealing temperature for 1 min, 72 °C for 1 min, and at 72 °C for 5 min, final extension using Takara Ex Taq (Takara Bio. Inc. Otsu, Japan).

### RNA sequencing and determination of transcript levels

The samples of developing xylem were collected using RNA Later (QIAGEN, Hilden, Germany) at the time of 9–11 am of April 22nd, 2015 followed by the method as described previously [13]. Concentration of RNA samples were measured by QUBIT fluorometer (Thermo Fisher Scientific Inc., Waltham, MT) and confirmed by using a Bioanalyzer Chip DNA 1000 series II (Agilent, Santa Clara, CA). All the samples were normalized to 150 ng/μl. The extracted RNAs were used for preparation of the mRNAseq library as described previously [13]. RNAseq was performed by MiSeq (Illumina, San Diego, CA) using the 150 bp paired-end run and data analysis was carried out as described previously [13]. From five plants each of high and low WBD group, ten RNA bulk libraries were paired-end sequenced on one lane of an Illumina Miseq flowcell. In high WBD group, this yielded a total of 116 million reads, with individual library yields ranging from 21 to 29 million reads. And in low WBD group, this yielded a total 111 million reads, with individual library yielding 19 to 27 million reads. *E. grandis* annotation v1.1 in PHYTOZOME v8.0 was cited as the reference sequence (https://phytozome.jgi.doe.gov/pz/portal.html#!info?alias=Org_Egrandis). The datasets generated and analyzed during the current study are available in the DDBJ database accession number DRA005369. The RPKM (reads per kilobase of exon per million fragments) values were used for the estimation of transcript levels of each gene [14]. Genes of RPKM (< 10.0) were eliminated as low transcript levels. The numbers of genes (RPKM > 10) of each group were listed in Additional file 1: Table S1. A differentially transcribed gene was specified upon a T-test with a 99% confidence rate (cutoff of 0.01).

### qRT-PCR

qRT-PCR analysis was carried out as described previously [13]. First strand cDNA was synthesized from 10 μg of total RNA using PrimeScript™ RT reagent kit (Takara Bio Inc., Kyoto, Japan). The primers used in this study are listed in Additional file 2: Table S2.

### Measurement of wood properties by near infrared (NIR) analysis

Wood cores containing developing xylem were removed 1.2 m above ground level. Each core was air-dried for predicting wood chemical composition, α-cellulose, hemicellulose and Klason lignin as described previously [13]. NIR spectroscopy was used to estimate wood chemical components. The air-dried wood was ground to pass through a 1-mm screen, and NIR spectra collected using NIRFlex (BUCHI, Switzerland) [13].

### Statistical analysis

We used SAS 9.2 software for data analysis of any significant differences between mean values. Correlation analysis was performed using Microsoft Excel statistical tools.

## Results

### Analysis of wood properties and selection of plant materials

Clone A was selected for a commercial plantation of Amapá Florestal e Celulose S.A. and its WBD was 587 kg/m^3^ (3.7 years) after planting. This clone also exhibited a good growth rate in northern Brazil. Open pollinated seeds of Clone A were collected in the orchard, germinated at the nursery and allowed to develop seedlings. A total of 102 seedlings of Clone A progeny were planted at 3.0 × 3.0 m spacing. The diameter at breast height (DBH) and tree height were measured for ninety-eight 2.4–2.8 year-old trees. Wood samples were also collected from these plants for analysis of wood properties. First, we investigated the distribution of WBD in the 98 progeny plants of Clone A. WBD was determined by NIR analysis using dried wood powders. Most of them had a WBD between 475 and 575 kg/m^3^ and the peak was observed at 525–550 kg/m^3^ (Fig. 1a). The numbers of progeny plants with over 575 kg/m^3^ or less than 450 kg/m^3^ of WBD were 8 and 10, respectively. At the same time, we evaluated the wood properties by NIR and predicted α-cellulose, Klason lignin and hemicellulose contents. We investigated the correlation between WBD and growth rate or wood components among the 98 progeny plants. Growth rate was measured by considering wood volumes, which were calculated by DBH and tree height. There was no correlation between WBD and growth rate (Additional file 3: Figure S1A). WBD did not also correlate with α-cellulose, hemicellulose and Klason-lignin contents (Additional file 3: Figure S1B-D). We then selected five progeny each plant with WBD of above 570 kg/m^3^ (H1-H5) or with less than 410 kg/m^3^ (L1-L5) for further analysis (Fig. 1b). Growth rates of 10 selected progeny plants were different from each other, with the low WBD group, L1-L5 exhibiting poor growth rate. On the other hand, in the high WBD group (H1-H5), H4 and H5 showed higher growth rate compared to the L group as well as the other members of the H group (Fig. 1c). Regarding wood compositions, no big differences were observed among the ten progeny plants (Fig. 1d). We carried out a SSR analysis to identify pollen parent (data not shown). Among ten plants, five were self-pollinated (H1, H2, H3, L2 and L5), another four (H4, L1, L3 and L4) were outcrossed and the other (H5) was unknown because the SSR analysis did not succeed. The possibilities of pollen parents of hybrid progeny plants were *E. grandis*, *E. urophylla* and *E. urophylla* x *E. grandis* F1 hybrid plants according to the lists of parent trees. There was no systematic difference in WBD and growth rate between selfed and outocrossed individuals.

### Cellulose biosynthesis

**Fig. 2:**
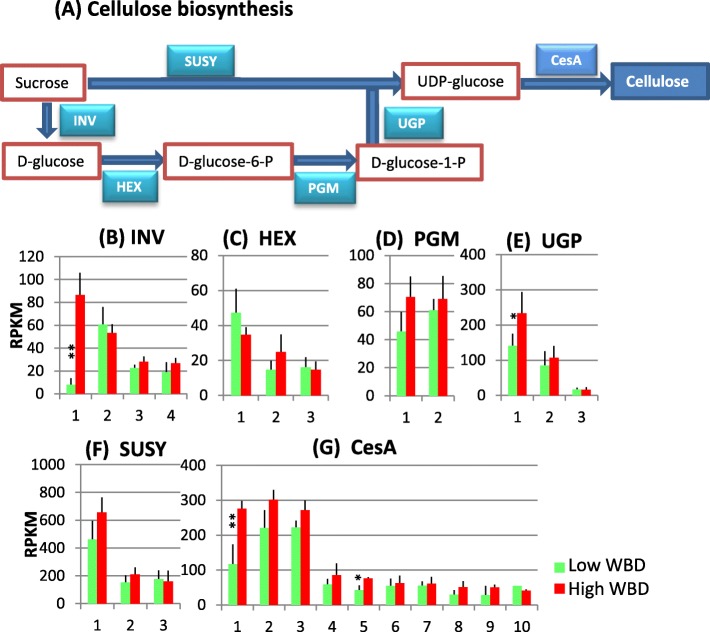
Cellulose biosynthesis pathways (**a**) and the transcript profiles of selected genes related to cell wall construction between the high and the low WBD types. Transcript levels of, INV (**b**), HEX (**c**), PGM (**d**), UGP (**e**), SUSY (**f**) and CesA (**g**) are shown. RPKM values are plotted on the Y-axis. Numbers on the X-axis represent gene numbers and *Arabidopsis* homolog gene names listed in Additional file 6: Table S3. The data collected from three technical replicates of each plant were analyzed. Mean values and standard deviations are shown with error bars. Asterisks or double asterisks indicate significant differences at *p* < 0.05 or *p* < 0.01, respectively

Cellulose is a major component in plant cell walls and is produced by cellulose synthase (CesA) from UDP-glucose that is biosynthesized via two different pathways: through sucrose synthase (SUSY) or through invertase (INV) (Fig. 2a). SUSY catalyzes the reversible conversion of sucrose into fructose and UDP-glucose. In several studies, an increase in SUSY expression levels was correlated with an increase in cellulose content [15, 16]. In another pathway, UDP-glucose is synthesized from D-glucose; sucrose is also hydrolyzed to D-glucose and fructose by INV enzymes. D-glucose is phosphorylated by hexokinase (HEX) using ATP as a phosphoryl donor and phosphoglucomutase (PGM) transfers the phosphate group on a D-glucose monomer from 1′ to the 6′ position in the forward direction or from 6′ to the 1′ position in the backward direction. UDP-glucose pyrophosphorylase (UGP), an important regulatory enzyme catalyzes the reversible reaction from glucose 1-phospate to uridine triphosphate to uridine diphosphate glucose and pyrophosphate [17]. In Eucalyptus, cellulose biosynthesis-related genes have been identified [18]. In the present study, we found that INV1, HEX2, PGM1&2 and UGP1 showed relatively higher expression in high WBD plants (Fig. 2). Three major transcribed SUSY genes were identified in hybrid *Eucalyptus* and SUSY1 showed strong expression in high WBD plants (Fig. 2f). There were ten CesA genes with relatively strong expression. Among them, three major CesA1–3 genes strongly transcribed in high WBD plants (Fig. 2g).

### Xylan biosynthesis

**Fig. 3:**
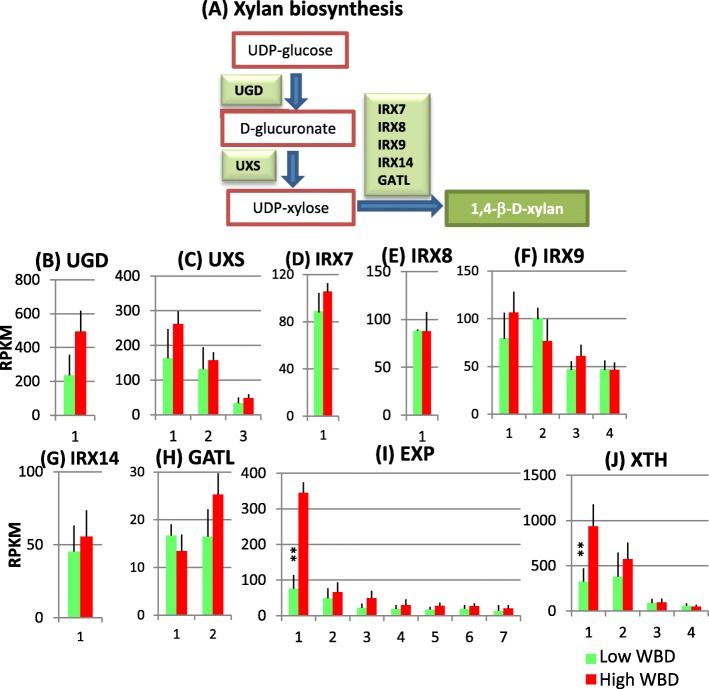
Xylan biosynthesis pathways (**a**) and the transcript profiles of selected genes related to cell wall construction, UGD (**b**), UXS (**c**), IRX7 (**d**), IRX8 (**e**), IRX9 (**f**), IRX14 (**g**), GATL (**h**), EXP (**i**) and XTH (**j**). Numbers on the X-axis represent gene numbers and *Arabidopsis* homolog gene names listed in Additional file 6: Table S3. RPKM values are plotted on the Y-axis. The data collected from three technical replicates of each plant were analyzed. Mean values and standard deviations are shown with error bars. Double asterisks indicate significant differences at *p* < 0.01

Xylan is a major polysaccharide in woody plants and is composed of a β-(1, 4)-linked xylose backbone with various substitutions. Xylan is synthesized by enzymes in the Golgi apparatus. The precursor of xylan biosynthesis is UDP-xylose, which is transferred from UDP-glucose by UDP-glucose dehydrogenase (UGD) and UDP-xylose synthase (UXS) (Fig. 3a). Genetic and biochemical analyses in *Arabidopsis* show that several genes, such as *irregular xylem*, IRX7, IRX8, IRX9, IRX14 and galacturonosyl transferase-like (GATL), are involved in xylan biosynthesis [18]. The genes involved in this process have also been documented previously [18]. Transcript level of UGD and three UXS genes were elevated in high WBD plants (Fig. 3b, c). IRX7 and IRX14 also showed a pattern of elevated levels of transcripts in high WBD plants (Fig. 3d, h). Among the plant cell wall-modifying genes, increased transcript levels of several expansin (EXP) and xyloglucan endo-transglycosylase/hydrolase (XTH) genes in high WBD plants were also found (Fig. 3i, j).

### Lignin biosynthesis

**Fig. 4:**
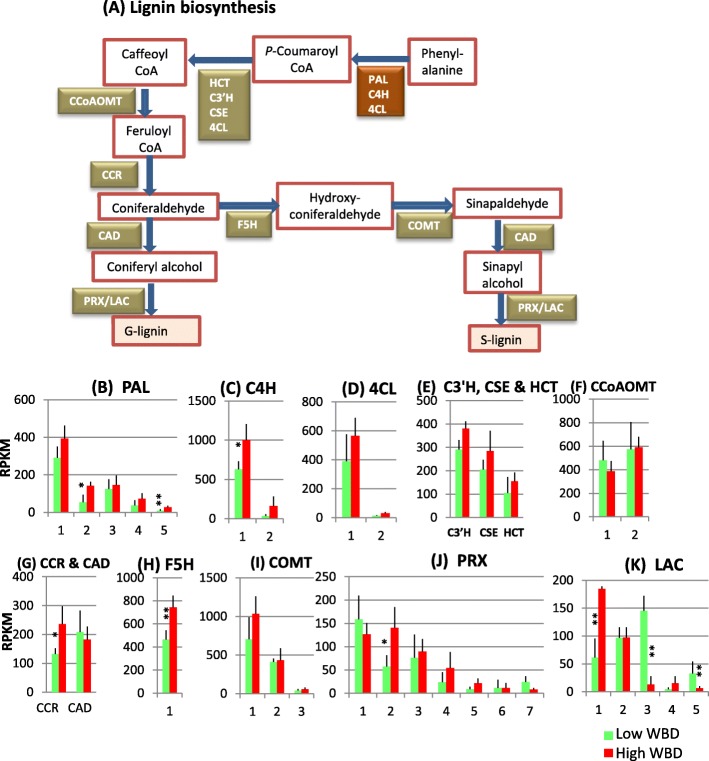
Lignin biosynthesis pathway (**a**) and comparison of the transcript profiles of genes related to lignin biosynthesis, PAL (**b**), C4H (**c**), 4CL (**d**), C3’H, HCT and CSE (**e**), CCoAOMT (**f**), CCR and CAD (**g**), F5H (**h**), COMT (**i**), PRX (**j**) and LAC (**k**). Numbers on the X-axis represent gene numbers and *Arabidopsis* homolog gene names listed in Additional file 7: Table S4. RPKM values are plotted on the Y-axis. Data collected from three technical replicates of each plant were analyzed. Mean values and standard deviations are shown with error bars. Asterisks or double asterisks indicate significant differences at *p* < 0.05 or *p* < 0.01, respectively

Three types of lignin polymers are reported, *p*-hydroxyphenyl (H) lignin that is polymerized from *p*-coumaryl alcohol, guaiacyl (G) lignin polymerized from coniferyl alcohol, and syringyl (S) lignin polymerized from sinapyl alcohol. Monolignol biosynthesis is started from phenylalanine to hydroxycinnamoyl-CoA esters through the common phenylpropanoid pathway (Fig. 4a). In *Eucalyptus*, 11 enzymes, phenylalanine ammonia lyase (PAL), cinnamate 4-hydroxylase (C4H), 4-coumarate CoA ligase (4CL), hydroxycinnamoyl CoA:shikimate hydroxycinnamoyl transferase (HCT), *p*-coumaroyl shikimate 3'-hydroxylase (C3’H), caffeoyl shikimate esterase (CSE), caffeoyl CoA *O*-methyltransferase (CCoAOMT), ferulate 5-hydroxylase (F5H), caffeic acid/5-hydroxyferulic acid 3/5 *O*-methyltransferase (COMT), cinnamoyl CoA reductase (CCR) and cinnamyl alcohol dehydrogenase (CAD) are involved in catalyzing monolignol biosynthesis [19]. As shown in Fig. 4b-d, PAL, C4H, and 4CL genes exhibited strong expression in the high WBD plants. Transcript levels of C3’H, CSE and HCT that catalyze the conversion of *p*-coumaroyl CoA to caffeoyl CoA were also high in high WBD plants (Fig. 4e). Two methylation steps by CCoAOMT and COMT are existed in monolignol biosynthesis pathway [20]. CCoAOMT1, 2 and COMT2, 3 genes showed no difference in transcript levels between the two groups (Fig. 4f, i). F5H is involved in the pathway leading to S lignin and catalyzes the step to produce hydroxyconiferaldehyde from coniferaldehyde. There is only one F5H gene transcribed in xylem tissue and a significantly strong transcript level of F5H was found in high WBD plants (Fig. 4h). CCR also showed higher level of transcript abundances in high WBD plants but the CAD gene showed similar transcript levels (Fig. 4g).

Monolignol polymerizing enzymes, peroxidase (PRX) and laccase (LAC) showed interesting results. PRX2, 3&4 exhibited relatively strong expression in high WBD plants compared to low WBD plants, while one major LAC1 in high WBD plants showed greater transcript level and another LAC3 showed higher transcription in low WBD plants (Fig. 4j, k).

### Cytoskeleton-related and reference genes

**Fig. 5:**
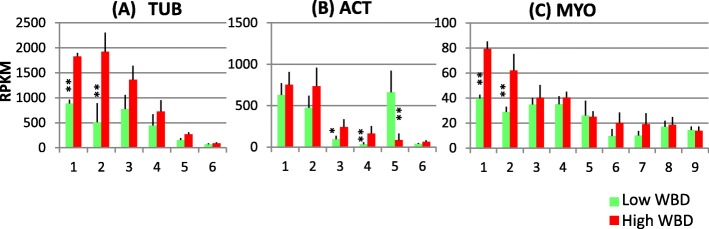
Transcript profiles of cytoskeleton genes, TUB (**a**), ACT (**b**) and MYO (**c**). Numbers on the X-axis represent gene numbers and *Arabidopsis* homolog gene names listed in Additional file 8: Table S5. RPKM values are plotted on the Y-axis. Mean values and standard deviations are shown with error bars. Asterisks or double asterisks indicate significant difference at *p* < 0.05 or *p* < 0.01, respectively

A total of 6 tubulin (TUB), 6 actin (ACT) and 9 myosin (MYO) genes were selected as cytoskeleton-related genes. Transcript levels of these cytoskeleton-related genes showed interesting patterns. While most of them exhibited higher levels in high WBD plants, ACT5 level was higher in the low WBD plants (Fig. 5a-c).

As reference genes, 10 ubiquitin (UBI) and 10 histone (HST) were investigated (Additional file 4: Figure S2). Ubiquitin is a small (8.5 kDa) protein that are found in almost all tissues in eukaryotic organisms [21]. Transcript abundances of most UBI and HST genes were similar in both plant groups. Therefore, these ten genotypes might not have different overall physiological conditions regarding with environmental stresses.

### Transcription factors

**Fig. 6:**
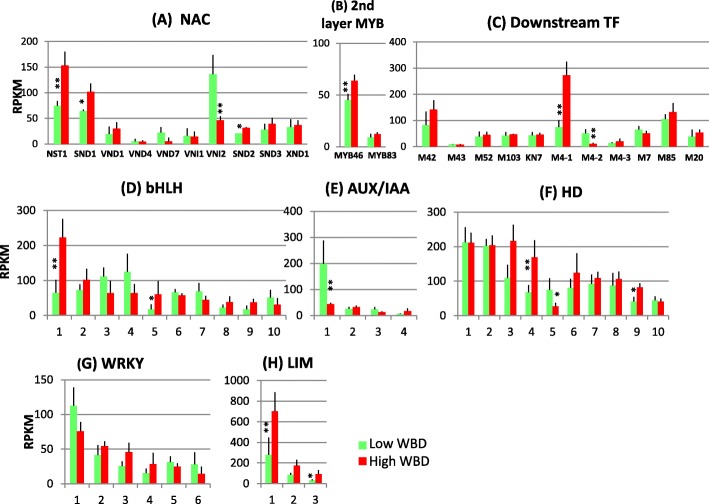
Comparison of the transcript profiles of transcription factor genes related to cell wall construction, NAC (**a**), 2nd layer MYB (**b**), downstream TF (MYB is abbreviated as M), (**c**), bHLH (**d**), AUX/IAA (**e**), HD (**f**), WRKY (**g**) and LIM (**h**). Numbers on the X-axis represent gene numbers and *Arabidopsis* homolog gene names listed in Additional file 9: Table S6. RPKM values are plotted on the Y-axis. The data collected from three technical replicates of each plant were analyzed. Mean values and standard deviations are shown with error bars. Asterisks or double asterisks indicate significant differences at *p* < 0.05 or *p* < 0.01, respectively

Several genetic and biochemical analyses conducted in the model plant *A. thaliana* have shown involvement of various transcription factors in secondary cell wall formation (for a review [22]). In *E. grandis*, more than 100 members of NAC secondary wall thickening promoting factor (NST) and MYB domain transcription factors have been shown [23, 24]. The NAC domain transcription factors, NST1&2, Secondary wall-associated NAC domain protein (SND)1 and vascular-related NAC-domain (VND)1–7, play a crucial role as the primary layer master switch in secondary cell wall formation. The secondary layer master switches are MYB46 and MYB83. Many MYB and KNOTTED-like from *Arabidopsis thaliana* (KNAT)7 proteins act as downstream transcription factors [17, 22]. The transcript levels of orthologous genes in hybrid *Eucalyptus* were investigated. Transcript levels of several orthologous NST1 and SND1 genes were significantly elevated in high WBD plants, whereas the level of VND-interacting (VNI)2, which is a transcriptional repressor of VND7 in *Arabidopsis*, was higher in low WBD plants (Fig. 6a). However, MYB46 also showed higher transcript levels in high WBD plants (Fig. 6b). In downstream transcription factors, MYB4–1 gene highly transcribed in high WBD plants, whereas MYB4–2 transcript was significantly lower (Fig. 6c). MYB4 acts a repressor of lignin biosynthesis. Transcript levels of other MYB genes and KNAT7 gene were almost the same between the two groups (Fig. 6c).

Several genes encoding the bHLH transcription factor, Auxin/Indoleacetic acid (AUX/IAA), Homeodomain (HD), WRKY, and LIM are involved in wood formation. Many of their isogenes are expressed in developing xylem tissues and their transcript levels were measured (Fig. 6d-h). Two bHLH1&5 genes demonstrated significantly positive expression in high WBD plants (Fig. 6d). The expression levels of AUX/IAA, HD and WRKY transcription factors looked almost the same in both groups although there were a few differences, especially with the AUX/IAA1 gene and HD5 gene, which showed significantly higher expression in the low WBD plants (Fig. 6e, f). LIM1–3 transcription factor genes transcribed higher in high WBD plants (Fig. 6h).

### qRT-PCR to validate transcript levels

We measured relative transcript levels of the ten genes, CesA2, CesA3, 4CL1, HCT, CSE, F5H, NST1, bHLH1, HD8 and LIM1 by qRT-PCR with UBI1 and HST1 genes as reference genes to validation of RNA-seq. The relative transcript levels of these ten genes with both reference genes obtained from qRT-PCR experiments showed similar values to those obtained from RNA-seq experiments (Additional file 5: Figure S3). The qRT-PCR experiment results confirmed the reliability of the RNA-seq data obtained from this study.

## Discussion

### Correlation between WBD and wood composition

WBD is a crucial trait for kraft pulp production as it impacts specific wood consumption. Especially since the Japanese pulp and paper industries import wood chips from abroad (from the Southern hemisphere), wood chips with high WBD offer great advantage for the Japanese pulp industry. Generally, slow growing trees show higher WBD in angiosperm plant species [25, 26]. In *Eucalyptus*, there are some investigations showing negative correlation between WBD and DBH [27–29]. However, some reports have also shown no relation between them [30, 31]. Here, we investigated if there were any correlations between WBD and wood volume as well as with wood compositions (α-cellulose, hemicellulose and Klason lignin) of open-pollinated 98 progeny plants of the *Eucalyptus* plant Clone A. WBD did not show any correlation with wood volume or wood compositions (Additional file 3: Figure S1). Studies in populations of poplar hybrids have shown a negative correlation of biomass growth (usually measured as wood volume) and lignin content [32, 33]. *Eucalyptus* half-sib families used in this study exhibited no correlation between WBD and lignin content. This might be caused by the plant materials used in this study, a single half-sib family where meiotic recombination of interspecific alleles would generate wide segregation and independent segregation of major gene effects for the two traits.

### Transcript levels of lignocellulose biosynthesis genes: Cellulose, xylan and lignin

We collected RNA samples from developing xylem tissues of each tree on April, 2015. April is rainy season in Amapá state, Brazil. Average monthly rainfall there on April is 380 mm (https://weather-and-climate.com/average-monthly-precipitation-Rainfall,Macapa,Brazil). Therefore, it is expected good growing season for Eucalyptus trees planted there. The xylem transcriptome analysis of lignocellulose biosynthesis-related genes reveals that many genes involved in cellulose, xylan and lignin biosynthesis transcribed strongly in high WBD plants (Figs. 2, 3 and 4). Especially, transcript levels of INV1, UGP1 and CesA1–3 genes that are involved in cellulose biosynthesis, UGD and UXS1 genes involved in xylan biosynthesis, and EXP1 and XTH 1 involved in cell wall modification were higher in high WBD plants (Figs. 2, 3). In addition, PAL2&5, C4H1, 4CL1, C3’H, CSE, CCR and F5H genes involved in monolignol biosynthesis also transcribed at increased levels in high WBD plants (Fig. 4). The transcript levels of genes encoding the cell wall modifying enzymes EXP and XTH also showed higher levels of expression in high WBD plants (Fig. 3). There are several reports concerning gene expression comparison of high and low WBD groups in *Eucalyptus* species, its hybrid, and *Pinus radiata* [2, 9, 10]. In pine, CesA and SUSY genes strongly transcribed in juvenile wood of high WBD while PRX and XTH were at higher levels in low WBD plants [10]. In *E.globulus*, the expression of PAL, CAD, 4CL and F5H genes was analyzed in genotypes with contrasting WBDs. In both groups, transcript abundances for CAD, PAL, and 4CL were similar and only F5H presented significant differences between the groups [9]. High transcript level of F5H gene in high WBD plants may have a potential to lead high syringyl/guaiacyl ratio but we have not measured it. No significant differences in transcript levels of the genes involved in lignocellulose biosynthesis was reported by RNA-seq analysis of 472 genes in hybrid *Eucalyptus* with contrasting WBD [2]. Our results clearly suggest a correlation between the thick secondary cell walls observed in high WBD plants and elevated transcript levels of lignocellulose biosynthesis related-genes as compared to those in low WBD plants.

### Cytoskeleton-related genes

Transcript levels of several genes belonging to the cytoskeleton, TUB, ACT and MYO were investigated in both plant groups for the evaluation of whole transcription levels (Fig. 5). Most transcript levels of TUB, ACT and MYO genes demonstrated significantly or relatively higher transcript levels in high WBD plants (Fig. 5). The cytoskeleton provides inner support for plant cells [34]. TUB proteins are components of microtubules. ACT is a major component of the plant cytoskeleton. MYO proteins are the molecular motors that use the chemical energy stored in ATP and convert it into a mechanical displacement. Indeed, the cytoskeleton including microtubules and actin filaments, can regulate cell wall deposition in several ways. In one way, the cortical microtubules define the insertion sites of CesA complexes and guide the direction of these complexes during cellulose synthesis in growing plant cells [35, 36]. As cellulose mainly contributes to cell wall strength, the orientation of the microtubule array substantially gives to the anisotropy of the cell walls. Thus, cellulose and microtubules provide cell growth perpendicular to their net orientation. In our study, transcript levels of most CesA genes as well as other cytoskeleton-related genes, such as TUB, ACT and MYO, were higher in high WBD plants. These results suggest that cytoskeleton formation may be associated with plant cell wall formation, especially cellulose biosynthesis.

### Transcription factors

Deposition of secondary cell wall during wood formation requires coordinated expression of genes involved in secondary cell wall biosynthesis, which is regulated by a secondary cell wall transcriptional network conserved in vascular plants [17]. In this transcriptional network, secondary cell wall NAC transcription factors are first layer master switches that can turn on the entire secondary cell wall formation. In *Arabidopsis*, ten NAC domain transcription factors, NST1&2, SND1 and VND1–7 regulate secondary cell wall biosynthesis [22]. In *Eucalyptus*, a report analyzed the structural, evolutionary and functional aspects of the NAC domain transcription factors [23]. In our study, three NAC genes, NST1, SND1, and VND2 transcribed strongly and the transcript levels of NST1 and SND1 genes were higher in high WBD plants, as shown in Fig. 6a.

MYB46 and MYB83 act as the second layer master switches regulating secondary cell wall biosynthesis [37, 38]. MYB46 and MYB83 are expressed in both vessels and fibers, and the phenotype of the simultaneous mutations of MYB46 and MYB83 was the loss of secondary cell wall thickening in vessels and fibers [38]. Similar to the first layer master switch, NST1 and SND1 were able to induce the transcription of cellulose, xylan and lignin biosynthesis genes [37, 38]. Structural and functional analysis of *E. grandis* MYB transcription factors have also been reported [24]. In our study, transcript level of the MYB46 was high in high WBD plants (Fig. 6b). These results indicate the possibility of the existence of the same mechanism involving secondary cell wall formation between the herbaceous plant *Arabidopsis* and the woody plant *Eucalyptus*.

A number of downstream transcription factors, *Arabidopsis* SND2, SND3, xylem NAC domain (XND)1, MYB4, MYB7, MYB20, MYB42, MYB43 MYB52, MYB85, MYB103 and KNAT7 have been shown to play crucial roles in regulating secondary cell wall biosynthesis [17]. These transcription factors act as activators or repressors of secondary cell wall biosynthesis or lignin specific biosynthesis. *Arabidopsis* MYB4 and MYB7 genes are probably negative regulators of lignin biosynthesis [17]. Here, *Eucalyptus* orthologous genes MYB4–1 and MYB4–2 transcribed with opposite patterns (Fig. 6c). Therefore, there is a possibility of a different role for each of these downstream regulators from those of *Arabidopsis* regulators. Regarding the other transcription factors, such as bHLH, AUX/IAA, HD and LIM family proteins, several genes exhibited significant transcript levels in high WBD plants (Fig. 6). LIM domain transcription factor was screened as a lignin biosynthesis-specific regulator that binds to the AC-rich region [39, 40]. Transgenic *E. camaldulensis* woody plants with antisense LIM, downregulated transcript levels of several genes such as *PAL*, C*4H* and *4CL* of the phenylpropanoid biosynthesis pathway [41–43]. Functional analysis including genetic or biochemical approaches would be needed for deciphering the precise physiological roles of these genes.

Here the possible correlation between the transcript levels of many genes involved in lignocellulose biosynthesis and WBD in ten hybrid *Eucalyptus* F2 progeny plants showing contrasting WBD values, was investigated. From comparisons of RNA levels and phenotypic analyses, we found that lignocellulose biosynthesis-related genes may be associated with WBD. Our previous study revealed that hybrid *Eucalyptus* trees with high lignin content showed higher transcript levels involved only in lignin biosynthesis [13]. These results indicate that trees with high WBD possess increased lignocellulose and transcript levels of lignocellulose biosynthesis-related genes. Which gene controls high WBD has been remained. One possible explanation is that *Arabidopsis* ortholog genes of first layer NAC domain transcription factors may regulate wood formation for high WBD. *Eucalyptus* plantation and breeding companies make a big effort for selection superior trees with higher growth rates, WBD and kraft pulp yield. Useful selection markers for higher WBD could be provided by indication that higher transcript abundances of lignocellulose biosynthesis-related genes. However, a question which gene as a trigger regulates high WBD has been still remained. Finally, this research provides a dataset of for selecting elite candidate trees by monitoring transcript levels of several lignocellulose biosynthesis-related genes.

## Conclusions

We investigated the correlation between WBD and wood volumes or wood properties using hybrid *Eucalyptus* progeny plants whose mother tree had 587 kg/m^3^ at 3.7 year-old of WBD. There were no correlations between WBD and wood volume or the components of wood, α-cellulose, hemicellulose and Klason lignin content. Transcriptome analysis of five plants each from two groups, a high- (570–609 kg/m^3^) or a low-WBD (378–409 kg/m^3^) group was carried out by RNA-Seq. We compared the transcriptional profiles of lignocellulose biosynthesis in plants with contrasting WBD content. Lignocellulose biosynthesis-related genes, such as CesA, INV, C4H and CCR showed higher transcript levels in high WBD group. Interestingly, strong transcript levels of several cytoskeleton genes encoding TUB, ACT and MYO were also observed in high WBD plants. In addition, we also found elevated transcript levels of genes encoding NAC, MYB, bHLH, HD and LIM transcription factors in high WBD plants. All these results show that high WBD could be the result of increased transcription of lignocellulose genes.

## Additional files

Additional file 1: **Table S1.** The total RNA-seq dataset (RPKM > 10). (XLSX 10 kb)

Additional file 2: **Table S2.** Gene-specific primers used in qRT-PCR. (XLSX 9 kb)

Additional file 3: **Figure S1.** Correlation between WBD and wood volume (A), α-cellulose content (B), Klason lignin content (C) and hemicellulose content (D). (PPTX 84 kb)

Additional file 4: **Figure S2.** Transcript profiles of reference genes, UBI (A) and HST (B). Numbers on the X-axis represent gene numbers listed in Table S4. RPKM values are plotted on the Y-axis. Mean values and standard deviations are shown with error bars. (PPTX 47 kb)

Additional file 5: **Figure S3.** Relative transcript levels of CesA2, CesA3, 4CL1, HCT, CSE, F5H, NST1, bHLH1, HD8 and LIM1 genes in the two plant groups measured by qRT-PCR analysis. UBI1 (A) and HST1 (B) genes were used as the reference gene. Double asterisks indicate significant difference at *p* < 0.01, respectively. (PPTX 55 kb)

Additional file 6: **Table S3.** List of cellulose and xylan biosynthesis pathway genes and mean values of their transcript levels in both plant groups. *Arabidopsis* homolog names are also described. (XLSX 19 kb)

Additional file 7: **Table S4.** List of lignin biosynthesis pathway genes and mean values of their transcript levels in both plant groups. *Arabidopsis* homolog names are also described. (XLSX 15 kb)

Additional file 8: **Table S5.** List of reference and cytoskeleton genes and mean values of their transcript levels in both plant groups. *Arabidopsis* homolog names are also described. (XLSX 16 kb)

Additional file 9: **Table S6.** List of transcription factors and mean values of their transcript levels in both plant groups. *Arabidopsis* homolog names are also listed. (XLSX 19 kb)

## Abbreviations

4CL: 4-Coumarate-CoA ligase
ACT: Actin
AUX/IAA: Auxin/ indoleacetic acid
bHLH: basic helix-loop-helix
C3'H: *p*-Coumaroyl-CoA 3'-hydroxylase
C4H: Cinnamate-4-hydroxylase
CAD: Cinnamyl alcohol dehydrogenase
CCoAOMT: Caffeoyl-CoA 3-*O*-methyltransferase
CCR: Cinnamoyl-CoA reductase
CesA: Cellulose synthase
COMT: Caffeic acid/5-hydroxyferulic acid 3/5 *O*-methyltransferase
CSE: Caffeoyl shikimate esterase
DBH: Diameter at breast height
F5H: Ferulate 5-hydroxylase
GATL: Galacturonosyl transferase-like
HCT: Hydroxycinnamoyl-CoA shikimate/quinate hydroxycinnamoyl transferase
HD: Homeodomain
HEX: Hexokinase
HST: Histone
INV: Invertase
IRX: Irregular xylem
KNAT: KNOTTED-like from *Arabidopsis thaliana*
LAC: Laccase
LIM: Lin-11, Isl-1, Mec-3-like
MYB: Myeloblastosis
MYO: Myosin
NAC: NAM, ATAF, and CUC
NIR: Near infrared
NST: NAC secondary wall thickening promoting factor
PAL: Phenylalanine ammonium lyase
PGM: Phosphoglucomutase
PRX: Peroxidase
QTL: Quantitative Trait Locus
RPKM: Reads per kilobase of exon per million fragments
SND: Secondary wall-associated NAC domain protein
SSR: Simple sequence repeats
SUSY: Sucrose synthase
TF: Transcription factor
TUB: Tubulin
UBI: Ubiquitin
UGD: UDP-glucose dehydrogenase
UGP: UDP-glucose pyrophosphorylase
UXS: UDP-xylose synthase
VND: Vascular-related NAC-domain
VNI: VND-interacting
XND: Xylem NAC domain
XTH: Xyloglucan endo-transglycosylase/hydrolase
