# Supplementary material for: RNA-seq analysis of lignocellulose-related genes in hybrid Eucalyptus with contrasting wood basic density
Source: BMC Plant Biol. 2018 Aug 6;18:156. doi: 10.1186/s12870-018-1371-9 (PMC6080517; doi:10.1186/s12870-018-1371-9)
Supplement: Supplementary file 3 — Figure S1. Correlation between WBD and wood volume (A), α-cellulose content (B), Klason lignin content (C) and hemicellulose content (D). (PPTX 84 kb) [file 12870_2018_1371_MOESM3_ESM.pptx]

## Slide 1
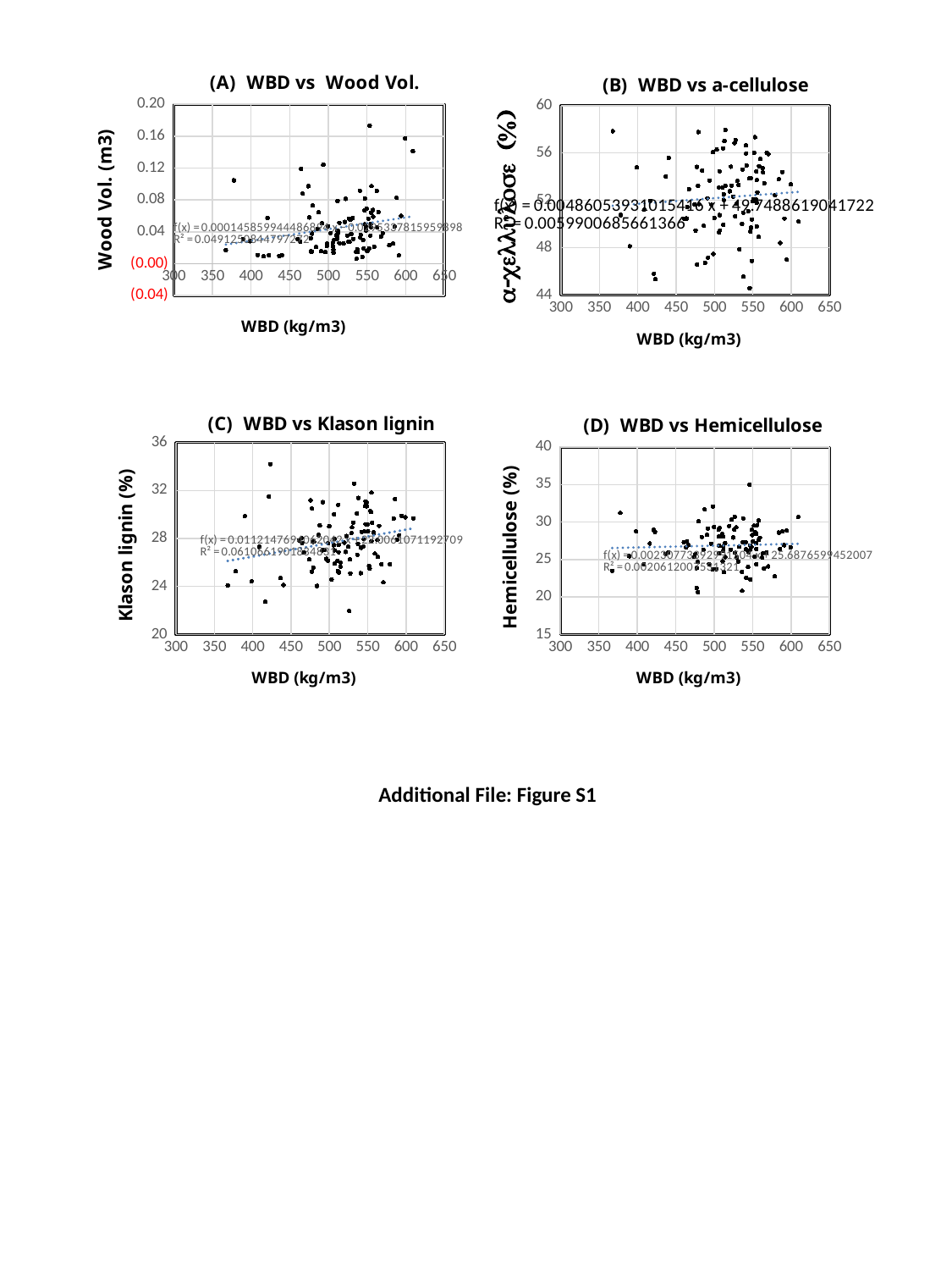

### Chart: (A) WBD vs Wood Vol.
| Category | |
|---|---|
### Chart: (B) WBD vs a-cellulose
| Category | |
|---|---|
### Chart: (C) WBD vs Klason lignin
| Category | |
|---|---|
### Chart: (D) WBD vs Hemicellulose
| Category | |
|---|---|Additional File: Figure S1
