# Supplementary figures and images for: RNA-seq analysis of lignocellulose-related genes in hybrid Eucalyptus with contrasting wood basic density
Source: BMC Plant Biol. 2018 Aug 6;18:156. doi: 10.1186/s12870-018-1371-9 (PMC6080517; doi:10.1186/s12870-018-1371-9)

## Slide 1
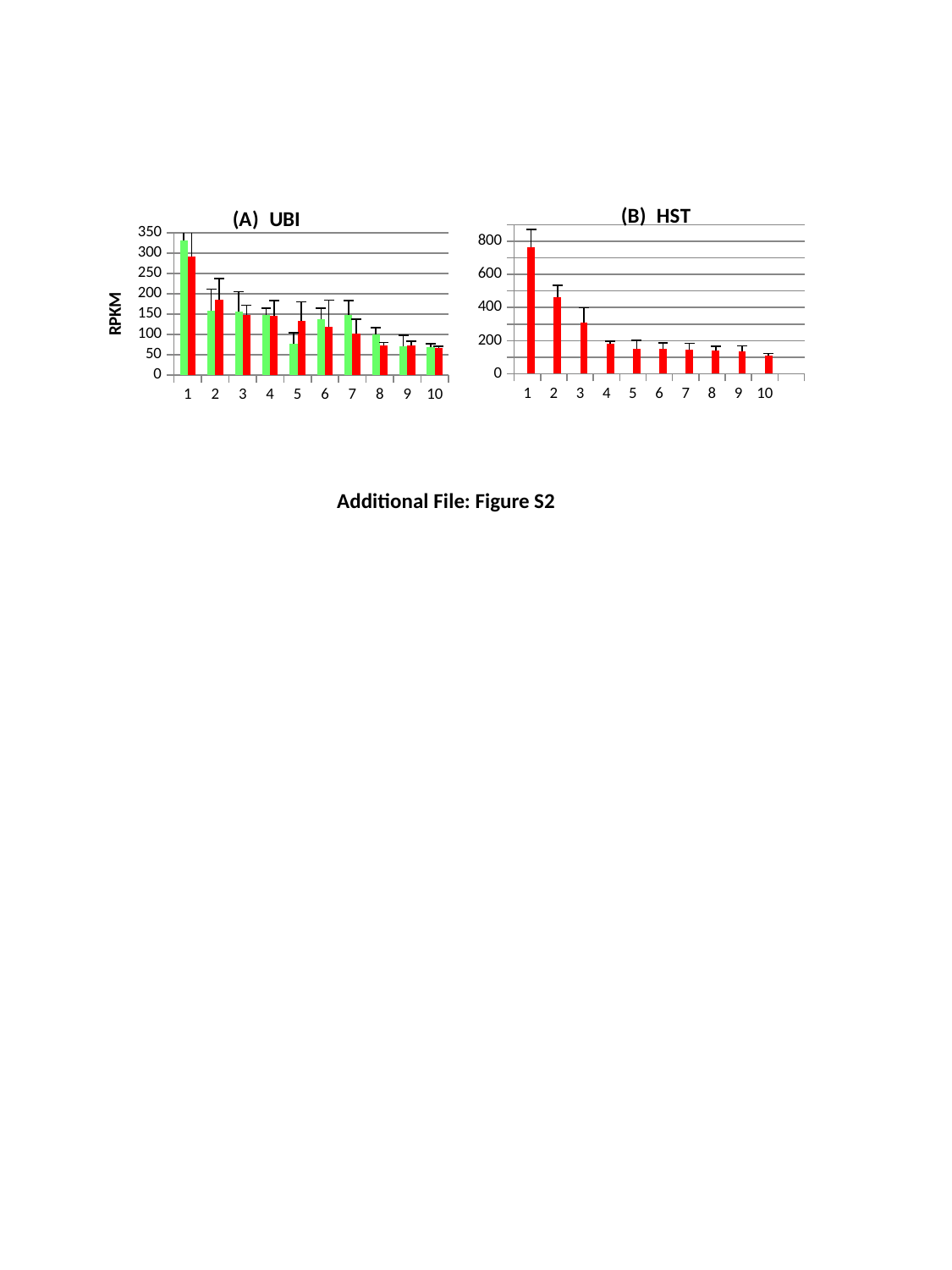

### Chart: (A) UBI
| Category | | |
|---|---|---|
### Chart: (B) HST
| Category | | |
|---|---|---|Additional File: Figure S2

Supplement: Supplementary file 4 — Figure S2. Transcript profiles of reference genes, UBI (A) and HST (B). Numbers on the X-axis represent gene numbers listed in Table S4. RPKM values are plotted on the Y-axis. Mean values and standard deviations are shown with error bars. (PPTX 47 kb) [file 12870_2018_1371_MOESM4_ESM.pptx]
