# Supplementary material for: RNA-seq analysis of lignocellulose-related genes in hybrid Eucalyptus with contrasting wood basic density
Source: BMC Plant Biol. 2018 Aug 6;18:156. doi: 10.1186/s12870-018-1371-9 (PMC6080517; doi:10.1186/s12870-018-1371-9)
Supplement: Supplementary file 5 — Figure S3. Relative transcript levels of CesA2, CesA3, 4CL1, HCT, CSE, F5H, NST1, bHLH1, HD8 and LIM1 genes in the two plant groups measured by qRT-PCR analysis. UBI1 (A) and HST1 (B) genes were used as the reference gene. Double asterisks indicate significant difference at p < 0.01, respectively. (PPTX 55 kb) [file 12870_2018_1371_MOESM5_ESM.pptx]

## Slide 1
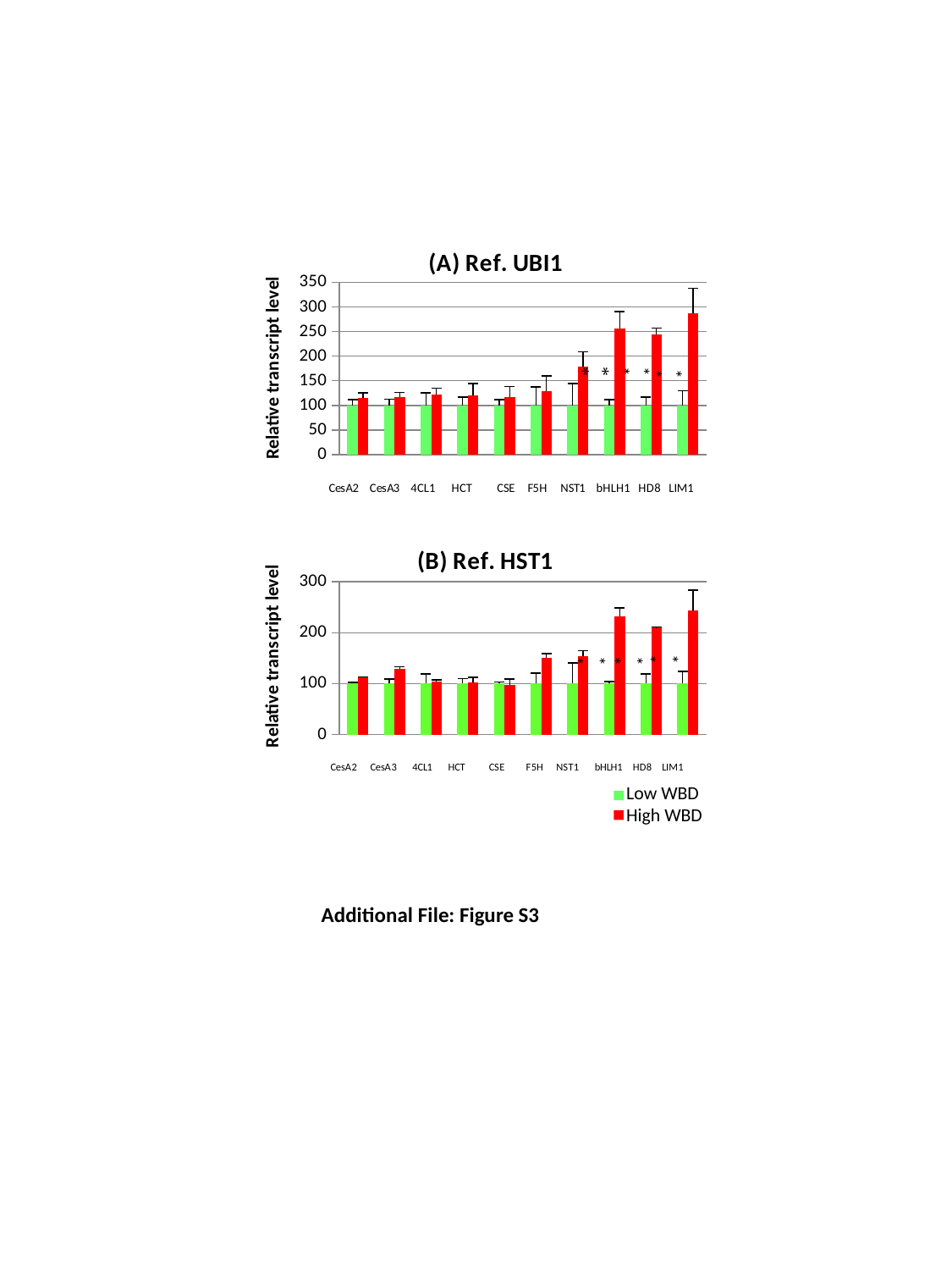

### Chart: (A) Ref. UBI1
| Category | L | H |
|---|---|---|
| CesA1 | 100.0 | 114.43372982075918 |
| CesA2 | 100.0 | 116.1828915222363 |
| 4CL1 | 100.0 | 121.48290994483315 |
| HCT1 | 100.0 | 120.72235633432136 |
| CSE1 | 100.0 | 116.15630970226985 |
| F5H1 | 100.0 | 128.49002788174784 |
| NAC1 | 100.0 | 178.6480973804768 |
| bHLH | 100.0 | 257.06520494049266 |
| HD4 | 100.0 | 244.38821172166718 |
| LIM1 | 100.0 | 286.6160857041803 |
### Chart: (B) Ref. HST1
| Category | | |
|---|---|---|**
**
**
Low WBD
High WBD
Additional File: Figure S3
